# Three-Dimensional Quantification of Bone Mineral Density in the Distal Femur and Proximal Tibia Based on Computed Tomography: In Vitro Evaluation of an Extended Standardization Method

**DOI:** 10.3390/jcm10010160

**Published:** 2021-01-05

**Authors:** Hugo Babel, Patrick Omoumi, Killian Cosendey, Hugues Cadas, Brigitte M. Jolles, Julien Favre

**Affiliations:** 1 Swiss BioMotion Lab, Department of Musculoskeletal Medicine, Lausanne University Hospital and University of Lausanne (CHUV-UNIL), CH-1011 Lausanne, Switzerland; killian.cosendey@chuv.ch (K.C.); brigitte.jolles-haeberli@chuv.ch (B.M.J.); julien.favre@chuv.ch (J.F.); 2 Service of Diagnostic and Interventional Radiology, Lausanne University Hospital and University of Lausanne (CHUV-UNIL), CH-1011 Lausanne, Switzerland; patrick.omoumi@chuv.ch; 3 Unité Facultaire d’Anatomie et de Morphologie (UFAM), University of Lausanne (UNIL), CH-1005 Lausanne, Switzerland; hugues.cadas@unil.ch; 4 Institute of Microengineering, Ecole Polytechnique Fédérale Lausanne (EPFL), CH-1015 Lausanne, Switzerland

**Keywords:** bone mineral density, knee, registration, osteoarthritis, osteoporosis, quantitative computed tomography, computational anatomy

## Abstract

While alterations in bone mineral density (BMD) are of interest in a number of musculoskeletal conditions affecting the knee, their analysis is limited by a lack of tools able to take full advantage of modern imaging modalities. This study introduced a new method, combining computed tomography (CT) and computational anatomy algorithms, to produce standardized three-dimensional BMD quantification in the distal femur and proximal tibia. The method was evaluated on ten cadaveric knees CT-scanned twice and processed following three different experimental settings to assess the influence of different scans and operators. The median reliability (intraclass correlation coefficient (ICC)) ranged from 0.96 to 0.99 and the median reproducibility (precision error (RMSSD)) ranged from 3.97 to 10.75 mg/cc for the different experimental settings. In conclusion, this paper presented a method to standardize three-dimensional knee BMD with excellent reliability and adequate reproducibility to be used in research and clinical applications. The perspectives offered by this novel method are further reinforced by the fact it relies on conventional CT scan of the knee. The standardization method introduced in this work is not limited to BMD and could be adapted to quantify other bone parameters in three dimension based on CT images or images acquired using different modalities.

## 1. Introduction

Quantifying the alterations in bone mineral density (BMD) is of interest in a number of conditions affecting the knee, including fractures [1,2,3], arthroplasty [4,5,6], meniscal damage and repair [7,8,9], osteoporosis [10,11], and osteoarthritis [12,13,14]. So far, in-vivo analysis of BMD has primarily relied on dual x-ray absorptiometry (DXA). However, the two-dimensional nature of DXA limits the assessment of spatial variations and the detection of localized alterations in BMD. To improve our understanding of the pathophysiological implications of BMD alterations, a primer to wider uses of BMD measures in clinical evaluation, there is a need for noninvasive methods allowing for more comprehensive assessment of BMD in the knee.

Computed tomography (CT) is an interesting alternative for the three-dimensional quantification of BMD [1,14,15,16,17]. The assessment of knee BMD is of particular interest [14,18], as radiation dose exposure with CT is not significant at this joint, contrary to other anatomical locations [19]. However, in order to conduct interpatient comparisons, there is a need to establish an anatomical correspondence between knees, as they naturally differ in size and shape. An anatomical correspondence is also necessary for longitudinal analyses in the case of pathologies that could alter the shape of the bones, such as osteoarthritis or tumors [20,21,22]. So far, the question of anatomical correspondence in the analysis of CT scans has generally been eluded by relying on regions of interest (ROIs) based on anatomical landmarks or geometrical guidelines [14,16,17,23,24,25,26]. However, the use of ROIs hinders the assessment of spatial variations in bone properties and does not take full advantage of CT scans by reducing information to a limited and low-resolution set of regional values.

Recently, computational anatomy methods were proposed to study bone structures [27]. These methods, firstly developed for brain analysis [28], register individual bones to a reference bone, thus allowing standardization of bone properties such as BMD. One such method was recently proposed for the proximal tibia [18]. However, as global changes in BMD have been observed in both the tibia and the femur in various pathologies [1,2,3,4,5,6,7,8,9,12,13,14], there is an interest to extend this method to the distal femur as well.

To ensure the suitability of BMD measures for clinical and research applications, the reproducibility and reliability of the BMD measures with respect to different scans and operators should be evaluated. To the authors’ knowledge, this evaluation, though essential, has seldom been undertaken in computational anatomy algorithms, particularly in those aiming to standardize BMD.

Thus, the study aimed to present a method to standardize BMD within the distal femur by extending an algorithm developed for the proximal tibia [18]. This work also aimed to evaluate the reproducibility and reliability of the proposed BMD standardization method.

## 2. Materials and Methods

### 2.1. Experimental Setup

Ten formalin-fixed cadaveric adult knees were scanned with CT and rescanned after repositioning. The acquisition was performed on a 40-row detector helical CT scanner (Discovery CT750HD; GE Medical Systems) using the following parameters: tube voltage, 120 kVp; reference tube current-time product, 200 mAs; and bone convolution kernel (U70u), voxel size of 0.5 × 0.5 × 0.312 mm. A solid calcium hydroxyapatite-based bone mineral reference phantom (Mindways Software, Austin, TX, USA) was used to establish a correspondence between the CT units and volumetric BMD (mg/cc). The knees had been stored in a refrigerator for up to 48 months before being scanned for this study. The research protocol was approved by the local ethics committee, and following local regulations regarding research on deceased persons, no demographic data was available for the samples. A senior musculoskeletal radiologist with more than 10 years of experience read the CT scans and, based on the presence and severity of osteophytes [29], concluded that five of the knees had degenerative changes (three mild and two severe changes).

### 2.2. Segmentation and Registration

**Figure 1:**
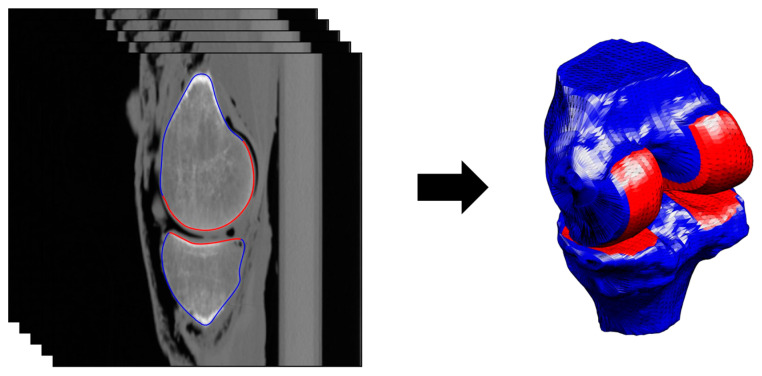
Segmentation of the femoral and tibial bones and identification of the subchondral bone areas (**left**), and resulting three-dimensional femoral and tibial bone meshes (**right**). In both plots, the subchondral bones are in red and the non-subchondral bones are in blue.

The tibial and femoral bones were segmented in the CT images using custom semi-manual segmentation tools [17,30], yielding three-dimensional triangular tibial and femoral bone meshes (Figure 1).

To describe the registration procedure, this paper uses the convention of moving and reference bones, with the moving bone being registered to the reference bone. Similar to a prior method for the proximal tibia [18], the registration involves two phases: (1) registering the surface of the moving bone to the surface of the reference bone (Section 2.2.1), followed by (2) propagating the surface-to-surface transformations to the CT voxels within the moving tibia and femur (Section 2.2.2). Following these two phases, both the surface of the bones and the BMD information contained within the image are registered to the reference bones.

#### 2.2.1. Phase 1

**Figure 2:**
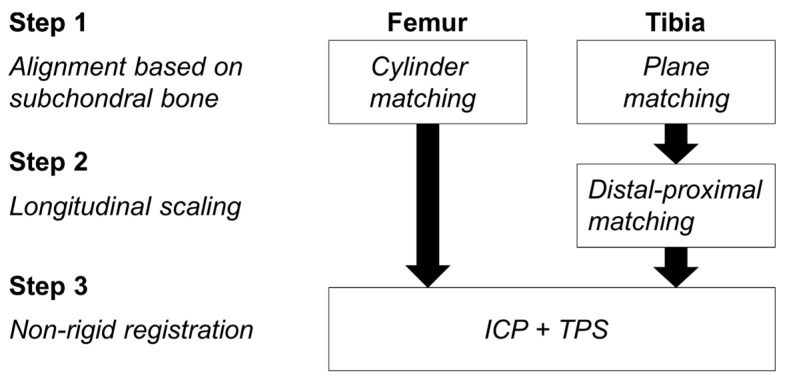
Flowchart of the first phase consisting of bone surface registration. ICP: Iterative Closest Point, TPS: Thin Plate Splines.

For the proximal tibia, the surface (mesh) of the moving bone was registered to the reference bone following a previously described method [18], involving three steps (Figure 2). First, the moving tibia is aligned to the reference tibia by combining a translation, a rotation, and an isotropic scaling based on the medial and lateral subchondral bone areas. Second, the moving tibia is scaled along its longitudinal axis to match the reference tibia. Third, the moving tibia is deformed locally using nonrigid registration to closely match the bone surface of the reference tibia.

**Figure 3:**
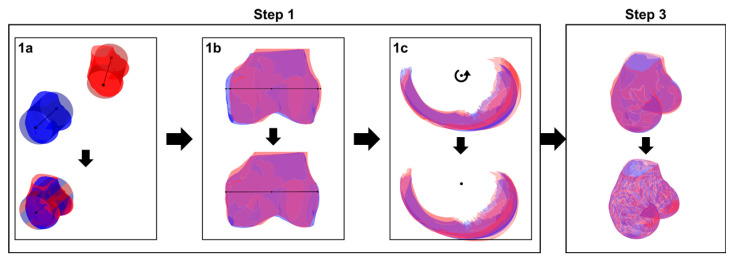
Illustration of the first phase of the registration procedure for the distal femur (see Figure 2 for an overall Figure 1. (**a**): the cylinder fitted to the subchondral bone area of the moving femur is aligned and scaled to coincide with the cylinder of the reference femur. Step 1. (**b**): the moving femur is scaled around and along the cylinder axis to match the size of the reference femur. Step 1. (**c**): the moving femur is rotated around the cylinder axis in order to align its subchondral bone area to the subchondral area of the reference femur. Step 2: not necessary for the femur. Step 3: a nonrigid transformation is applied to the moving femur to locally match the reference femur.

For the distal femur, the method in [18] was modified as follows. The first step of the registration procedure was adapted by fitting a cylinder to the subchondral bone area [30,31,32] of the moving and reference femurs (Figure 3, step 1a). After manual identification of the trochlear notch on both femurs, the moving femur was translated and rotated to align its cylinder axis and the projection of its trochlear notch on the cylinder axis to the cylinder axis and projection of the trochlear notch of the reference femur (Figure 3, step 1a). Then, the moving femur was scaled such that the radius of the two cylinders coincide and the condylar regions, from the trochlear notch to the medial and lateral epicondyles, were scaled separately along the cylinder axis to account for their individual sizes (Figure 3, step 1b). Next, the subchondral areas of the moving and reference femurs were aligned by rotating the moving femur around the cylinder axis to minimize the distance between subchondral areas (Figure 3, step 1c). This first step was necessary to limit differences between the two femurs in terms of bone size and placement in the scanner and to account for differences in orientation within the field of view of the scanner. The second step in the tibial registration procedure, which consisted in scaling the proximal tibia along its longitudinal axis to account for the high level of symmetry in the tibia, was not necessary for the distal femur (Figure 2). Finally, in the third step, the same method as previously described for the tibia, which combines nonrigid iterative closest point (ICP) [33] and thin-plate splines (TPS) [34], was used to locally deform the moving bone such that its surface closely matched the surface of the reference bone (Figure 3, step 3).

#### 2.2.2. Phase 2

During the second phase, the surface-to-surface transformations calculated in the first phase were applied to the voxels of the moving tibia and femur, as previously described for the tibia [18]. With this operation, the CT voxels were translated, scaled, rotated, and deformed.

### 2.3. Evaluation of the Method

For each cadaveric knee, the images from the (first) CT scan were segmented by two operators and the images from the rescan were segmented by one of the two operators, resulting in three sets of 10 segmented knees. The tibial and femoral bones resulting from each segmentation were then registered to a reference tibia and femur [18], following the procedure described above. Standardized BMD reporting was achieved through three-dimensional maps created by filling the reference tibia and femur with 7000 and 12,000 isotropic cells of 2 mm sides, respectively, and by determining the BMD value of each cell, for each segmentation, as the average of all registered CT voxels contained within the cell’s boundaries [18]. 

The reliability and reproducibility of the standardized BMD measures were evaluated in three settings: intra-operator/inter-scan, inter-operator/intra-scan, and inter-operator/inter-scan. The reliability was assessed for each of the 7000 tibial and 12,000 femoral cells by calculating the two-way random-effects intraclass correlation coefficient (ICC) [35] over the 10 knees for the repeated measurement. The reliability was classified as poor (ICC < 0.5), moderate (0.5 ≤ ICC < 0.75), good (0.75 ≤ ICC < 0.9), and excellent (ICC > 0.9) [36]. The reproducibility was assessed for each of the 7000 tibial and 12,000 femoral cells as the root mean square over the 10 knees for the precision error of the repeated measurement (RMSSD) [37]. The reliability and reproducibility were compared between settings using Wilcoxon signed-rank tests [38] with a Bonferroni correction for multiple comparisons, and the effect size was reported [39]. Lastly, the reproducibility and reliability maps were visually examined to assess local effects.

All processing in this study was done with custom software using Matlab R2019 b (Mathworks, Natick, MA, USA).

## 3. Results

**Figure 4:**
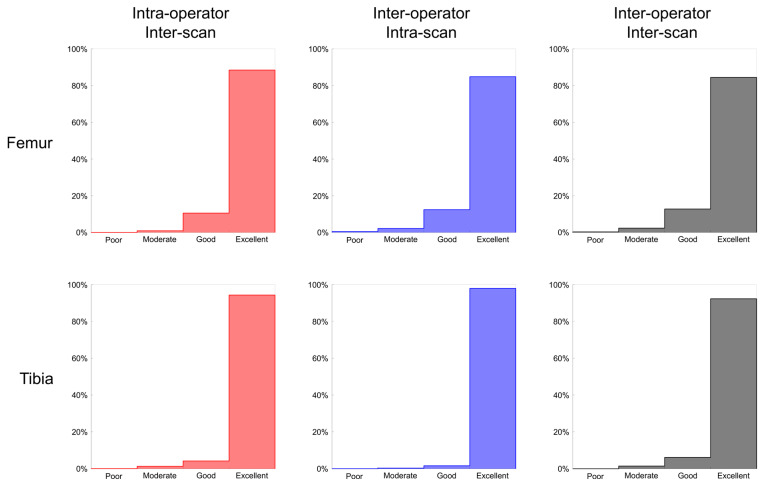
Histogram of the reliability for the 12,000 femoral (**top**) and 7000 tibial (**bottom**) cells in the three settings.

**Figure 5:**
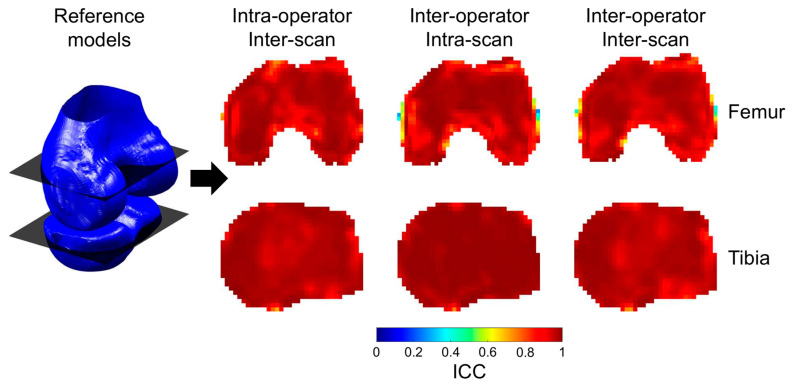
Illustration of the spatial variations in reliability: (**left**) reference distal femur and proximal tibia bone models with two representative coronal slices and (**right**) intraclass correlation (ICC) maps for the three settings at the coronal slices for the tibia (**top**) and the femur (**bottom**).

The median reliability of the 12,000 femoral cells ranged from 0.96 to 0.97 among the three experimental settings (Table 1), with more than 84% of the cells reporting an excellent ICC for each setting (Figure 4). The reliability in the inter-operator/inter-scan setting was statistically lower than in the two other settings (z ≤ −27.7, *p* ≤ 0.001), with a small effect size (≤0.29), and statistically higher in the intra-operator/inter-scan compared to the inter-operator/intra-scan setting (z = 4.0, *p* < 0.001), with a very small effect size (0.04). Two areas of lower reliability were observed within the femur for the two inter-operator settings: at the epicondyles and around the trochlear notch (Figure 5). The median reproducibility ranged from 9.56 mg/cc (2.5% of the BMD range) in the intra-operator/inter-scan setting to 10.75 mg/cc (2.8% of the BMD range) in the inter-operator/inter-scan setting (Table 1). The reproducibility in the inter-operator/inter-scan setting was statistically higher than in the two other settings (z ≥ 21.9, *p* ≤ 0.001), with a small effect size (≥0.33), and statistically higher in the inter-operator/intra-scan setting compared to the intra-operator/inter-scan setting (z = 5.7, *p* < 0.001), with a very small effect size (0.05).

In the tibia, the median reliability of the 7000 cells was excellent (ICC ≥ 0.97) for the three experimental settings (Table 1), with more than 92% of the cells having an ICC over 0.9 for each setting (Figure 4). The reliability was statistically higher in the inter-operator/intra-scan setting than in the two other settings (z ≥ 46.5, *p* < 0.001), with a medium effect size (≥0.56), and higher in the intra-operator/inter-scan setting than in the inter-operator/inter-scan setting (z = 32.8, *p* < 0.001), with a small effect size (0.39). The lower ICC were heterogeneously distributed among the cells. The median reproducibility ranged from 3.97 mg/cc (1.0% of the BMD range) in the inter-operator/intra-scan setting to 7.29 mg/cc (1.9% of the BMD range) in the inter-operator/inter-scan setting (Table 1). The reproducibility was statistically lower in the inter-operator/intra-scan setting than in the other two settings (z ≤ 41.2, *p* < 0.001), with a small to medium effect size (≥0.49), and lower in the intra-operator/inter-scan setting than in the inter-operator/inter-scan setting (z = −36.8, *p* < 0.001), with a small effect size (≥0.44).

## 4. Discussion

A method was presented to standardize BMD in the distal femur and proximal tibia with excellent reliability and adequate reproducibility to be used in clinical and research applications. This new possibility of comprehensive quantification of knee BMD in three dimension offers promising perspectives to improve our understanding of the role of BMD in the initiation and progression of musculoskeletal pathologies as well as to introduce novel BMD measures in clinics. In this regard, it is important to mention that the method reported in this work can be combined with existent techniques to automatically segment CT images [40,41], thus allowing standardized high-resolution reporting of knee BMD with minimal operator intervention.

While the reliability of the BMD standardization was excellent in all three settings, both for the femur and the tibia, there were local areas of lower reliability within the femur. Interestingly, lower reliability was observed in regions where segmenting the CT images was more delicate, such as peripheral regions around the epicondyles. Therefore, the lower reliability in these regions is most likely due to segmentation and not the registration procedure. Although differences in reliability among experimental settings achieved statistical significance, the reliability remained excellent for all three settings, suggesting that the standardization method can be used independently of the experimental conditions. No comparison with previous BMD standardization methods could be performed, as the reliability of femoral or tibial BMD has seldom been assessed in computational anatomy studies.

The reproducibility of the BMD standardization ranged from 4.0 to 10.7 mg/cc, which appears adequate with respect to the BMD differences, ranging from 33 to 150 mg/cc, previously reported with knee osteoarthritis or bone fractures [42,43,44,45,46]. Furthermore, the reproducibility reported in this study was consistent with data in the literature for proximal femoral BMD. Indeed, the reproducibility reported in Carballido-Gamio et al. [47] and Li et al. [48] for relatively large regions of interest (ROIs) ranges between 1.1 and 28 mg/cc. When attempting such a comparison across studies, it is important to note that the cells used in the present work are much smaller than the ROIs in Carballido-Gamio et al. and Li et al. and that, by definition, the reproducibility tends to be higher for larger volumes of interest [49,50]. Similar to the reliability results, reproducibility differences were observed among settings but were 3.1 to 37.5 times smaller than BMD differences reported in prior studies comparing control and pathological knees [42,43,44,45,46]. This further suggests that the standardization method is suitable independently of the experimental settings.

The present study has several strengths. First, the reliability and reproducibility were quantified on knees representative of the general population, suggesting that the results are applicable to a wide range of clinical presentations. Second, to the authors’ knowledge, this study is the first to assess comprehensively the reliability and reproducibility of a computational anatomy method to standardize BMD. The reliability and reproducibility data in the present work provide valuable information for future applications of the proposed method and for the field in general. Third, the method introduced in this work could be used to standardize other bone parameters, such as texture [51], or to assess the spatial distribution of bone defects, such as osteophytes, bone cysts, or bone marrow lesions, which are currently only assessed semi-quantitatively [52]. Fourth, the registration procedure establishes an anatomical correspondence based solely on bone surfaces. As such, it does not depend on the acquisition parameters or bone size. In addition, the registration procedure could be applied across imaging modalities.

This study also has limitations which should be discussed. First, the evaluation relied on cadaveric knees because subjecting individuals to repeated CT scans would have represented unnecessary and unethical risks [53]. An in vivo assessment of the method would be expected to yield similar results, as BMD differences between fresh and formalin-fixed cadavers have been reported to remain negligible, even after several months [54,55]. However, assessing the method with undocumented cadaveric specimens prevented evaluating the sensitivity of the method. Future applications of the method to longitudinal data or to knees of different conditions should therefore evaluate its capacity to detect changes or differences in BMD [37]. Further studies will also be necessary to compare BMD data obtained using the present method to data obtained using DXA. Additionally, although the evaluation relied on a small sample size, the number of knees was sufficient with respect to the study objectives and in the range of previous studies evaluating methods to quantify BMD at the hip joint [47,48].

## 5. Conclusions

In conclusion, this paper presented a method to standardize BMD in the distal femur and proximal tibia with excellent reliability and reproducibility. This method could find applications in both research and clinics. Specifically, it could contribute to novel BMD measures or be used to visualize and analyze location-specific three-dimensional BMD patterns.

## Figures and Tables

**Table 1:** Reliability and reproducibility of the bone mineral density (BMD) standardization.

	Intra-Operator Inter-Scan	Inter-Operator Intra-Scan	Inter-Operator Inter-Scan	
Femur			
Reliability ^#^	0.97 (0.94, 0.98)	0.97 (0.93, 0.98)	0.96 (0.93, 0.98)
Reproducibility ^#^	9.56 (7.10, 13.46)	9.67 (6.61, 14.68)	10.75 (8.16, 14.96)
Tibia			
Reliability *^,#^	0.97 (0.96, 0.98)	0.99 (0.98, 1.0)	0.97 (0.95, 0.98)
5	6.58 (5.70, 8.49)	3.97 (2.39, 7.39)	7.29 (5.92, 10.30)

Reliability (ICC) and reproducibility (RMSSD) data are presented as the median [1st quartile, 3rd quartile] over the 12,000 femoral or 7000 tibial cells. Reproducibility data are in mg/cc. Symbols indicate differences between experimental settings that achieved statistical significance (adjusted *p* < 0.05): * between the intra-operator/inter-scan and inter-operator/intra-scan settings, ^#^ between the intra-operator/inter-scan and inter-operator/inter-scan settings, and between the inter-operator/intra-scan and inter-operator/inter-scan settings.

## Data Availability

The data are not publicly available due to regulatory provisions.
